# Large‐scale genetic panmixia in the blue shark (*Prionace glauca*): A single worldwide population, or a genetic lag‐time effect of the “grey zone” of differentiation?

**DOI:** 10.1111/eva.12591

**Published:** 2018-02-22

**Authors:** Diane Bailleul, Alicia Mackenzie, Olivier Sacchi, François Poisson, Nicolas Bierne, Sophie Arnaud‐Haond

**Affiliations:** ^1^ IFREMER, UMR MARBEC, Station de Sète Sète France; ^2^ OREME – Station Marine Université Montpellier Sète France; ^3^ CNRS, Institut des Sciences de l'Evolution Université Montpellier Montpellier France

**Keywords:** blue shark, conservation, fisheries, genetic panmixia, *Prionace glauca*, stock

## Abstract

The blue shark *Prionace glauca*, among the most common and widely studied pelagic sharks, is a top predator, exhibiting the widest distribution range. However, little is known about its population structure and spatial dynamics. With an estimated removal of 10–20 million individuals per year by fisheries, the species is classified as “Near Threatened” by International Union for Conservation of Nature. We lack the knowledge to forecast the long‐term consequences of such a huge removal on this top predator itself and on its trophic network. The genetic analysis of more than 200 samples collected at broad scale (from Mediterranean Sea, North Atlantic and Pacific Oceans) using mtDNA and nine microsatellite markers allowed to detect signatures of genetic bottlenecks but a nearly complete genetic homogeneity across the entire studied range. This apparent panmixia could be explained by a genetic lag‐time effect illustrated by simulations of demographic changes that were not detectable through standard genetic analysis before a long transitional phase here introduced as the “population *grey zone*.” The results presented here can thus encompass distinct explanatory scenarios spanning from a single demographic population to several independent populations. This limitation prevents the genetic‐based delineation of stocks and thus the ability to anticipate the consequences of severe depletions at all scales. More information is required for the conservation of population(s) and management of stocks, which may be provided by large‐scale sampling not only of individuals worldwide, but also of loci genomewide.

## 1 INTRODUCTION

The peculiar life‐history traits of many marine species (e.g., large population sizes, high dispersal potential) often lead to weak or no genetic differentiation (Hedgecock, Barber, & Edmands, 2007; Waples, 1998). The accumulation of genetic differentiation theoretically depends on the number of migrants exchanged per generation (*N*_*e*_ *m*, with *N*_*e*_ the effective population size and *m* the rate of migration), whereas the level of demographic interdependency depends on the rate of migrants (*m*) exchanged (Lowe & Allendorf, 2010; Waples & Gaggiotti, 2006). In other words, the genetic connectivity and the demographic connectivity exhibit, in some conditions, a phase difference that prevents the former from being a good *proxy* of the latter. Common situations involving the homogeneous distribution of genetic polymorphism can thus derive from a wide range of very distinct demographic situations, depending on the relative weight of population size and effective dispersal. These demographic scenarios range from a rate of migratory exchange high enough to lead to both genetic homogeneity and strong demographic interdependency among (sub)populations, even with limited effective sizes, to nearly negligible rates of migratory exchange (*m*) among populations exhibiting large effective sizes (*N*_*e*_).

**Figure 1:**
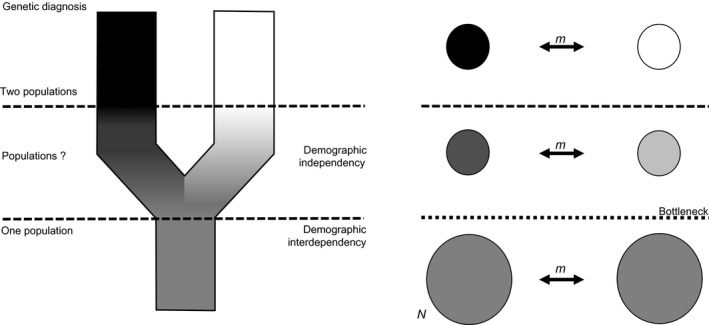
The “grey zone” of population differentiation. Analogy to De Queiroz speciation grey zone: inside the “grey zone”, it is impossible to discriminate populations based on genetic data alone. *N*, population size; *m*, migration rate

The incomplete genetic sorting of populations could be considered as the homologous version, at the intraspecific level of the “grey zone” from De Queiroz (2007). This “species grey zone” represents the lag during which, lineage sorting being incomplete, species delimitation is not possible based solely on the genetic information in hand. Here, we introduce the concept of “population *grey zone*.” In contrast to the “species grey zone” defined by De Queiroz, where an agreement on the actual number of species can be reached before the split or after a reasonable time of divergence, a consensus on the level of demographic connectivity (or interdependency) of populations estimated through the analysis of genetic connectivity can be reached only after the “population *grey zone*” (Figure 1).

Large pelagic species are often characterized by life‐history traits favouring this kind of observation, and the “population *grey zone*” concept may well be illustrated by the blue shark (*Prionace glauca*), the most abundant and widespread chondrichthyan (Nakano & Stevens, 2008). It has a worldwide distribution in temperate and tropical waters (Compagno, 1984; Nakano & Seki, 2003), with vertical occupation from the surface to 1,160 m (Queiroz, Humphries, Noble, Santos, & Sims, 2012). Blue sharks exhibit complex population movements, with seasonal migrations (Henderson, Flannery, & Dunne, 2001; Vandeperre et al., 2014) coupled with sexual and size segregation (Carrera‐Fernández, Galván‐Magaña, & Ceballos‐Vázquez, 2010; Kohler, Turner, Hoey, Natanson, & Briggs, 2002; Litvinov, 2006; Nakano & Stevens, 2008) as well as transatlantic (Kohler & Turner, 2008; Queiroz et al., 2005) and North–South migrations (Vandeperre et al., 2014). Conventional tagging and satellite studies have revealed high individual variability in movement, with the most mobile individuals tracked across tens of thousands of kilometres and up to 42 km per day, while the less mobile individuals may not exceed 18.8 km per day (Kohler & Turner, 2008; Queiroz et al., 2005; Vandeperre et al., 2014).

The blue shark is by far the most abundant shark species caught by fisheries (Beerkircher, Cortes, & Shivji, 2002; Buencuerpo, Rios, & Morón, 1998; Campana, Marks, Joyce, & Kohler, 2006; García‐Cortés & Mejuto, 2001; Rogan & Mackey, 2007). Most of the time, blue sharks are taken either as bycatches, that is, undesirable nontargeted species, by tuna longlines and swordfish fisheries (Carvalho et al., 2015) or as the targeted species, depending on the fishing location and the nationality of the fisheries. Indeed, Oliver, Braccini, Newman, and Harvey (2015) reported that “approximately 50% of the global shark production is composed of sharks caught as bycatch in the high seas pelagic longline fisheries” and Clarke, Harley, Hoyle, and Rice (2013) found that blue sharks caught in Pacific were either discarded (50%) or finned (42%). Although abundance studies have delivered mixed conclusions, the recent estimates most often converge towards a rather sharp decline in population during recent decades. The blue shark populations' trends could be estimated by CPUE (catch per unit effort), which is an indirect measure of the abundance of the species; CPUE variations signify changes to the species' real abundance. The blue shark CPUE seems to be static in the Atlantic (Nakano & Clarke, 2005), the Indian (Nakano, 1996 in Nakano & Stevens, 2008) and the Pacific Oceans (Matsunaga & Nakano, 1999). In contrast, Simpfendorfer, Hueter, Bergman, and Connett (2002) estimated an 80% male decline during 1980–1990 (females results lacked significance) in the North Atlantic, Baum et al. (2003) a 60% decline in the North Atlantic from CPUE and Ferretti, Myers, Serena, and Lotze (2008) a 97% decline in abundance in the Mediterranean Sea during the mid‐20th century. In the Pacific, blue sharks decline was estimated to have reached 57% during the period spanning from 1950 to 1990 (Ward & Myers, 2005) and 1995 to 2003 (Clarke et al., 2013). With an estimate of 10 million (Clarke et al., 2006) to 20 million individuals removed per year (Stevens, 2009), the blue shark is classified as *Near Threatened* worldwide and *Critically Endangered* in the Mediterranean Sea by the IUCN (International Union for Conservation of Nature, 2016). Many pelagic shark species exhibit philopatry (Barnett, Abrantes, Stevens, & Semmens, 2011; Feldheim et al., 2014; Hueter, Heupel, Heist, & Keeney, 2004; Jorgensen et al., 2010; Pardini et al., 2001). Blue shark mating, pupping and nursery sites are suspected in the Atlantic (Azores, Brazil and South Africa as nurseries, Aires‐da‐Silva, Ferreira, & Pereira, 2008; Vandeperre et al., 2014; Verissimo et al., 2017) and Pacific (California as nursery, Carrera‐Fernández et al., 2010; Caldera as pupping and nursery, Bustamante & Bennett, 2013) and Mediterranean Sea (as mating area and nursery, Megalofonou, Damalas, & de Metrio, 2009). Thus, despite long‐range migration, philopatry, together with recent reductions in population sizes, may lead to expect some level of genetic differentiation across the species range.

Specific challenges are associated with the study of pelagic and migrating sharks, particularly the estimation of population size and the delimitation of stocks of a worldwide species exploited by several nations and in international waters (Heist, 2008). The stock concept was initially proposed to address the sustainability of fishing activity (Carvalho & Hauser, 1994). Stock includes a broad range of definitions, depending on the management aims, and reconciling them is not always easy (Carvalho & Hauser, 1994). Fishery stock (Smith, Jamieson, & Birley, 1990) usually refers to a group of fishes exploited in a specific area or using specific gear, whereas biological stock (Ihssen et al., 1981) is defined as “an intraspecific group of randomly mating individuals with temporal and spatial integrity,” in line with the definition of a demographic population. The genetic stock, according to Ovenden (1990), is defined as “the largest group of animals that can be shown to be genetically connected through time.”

**Figure 2:**
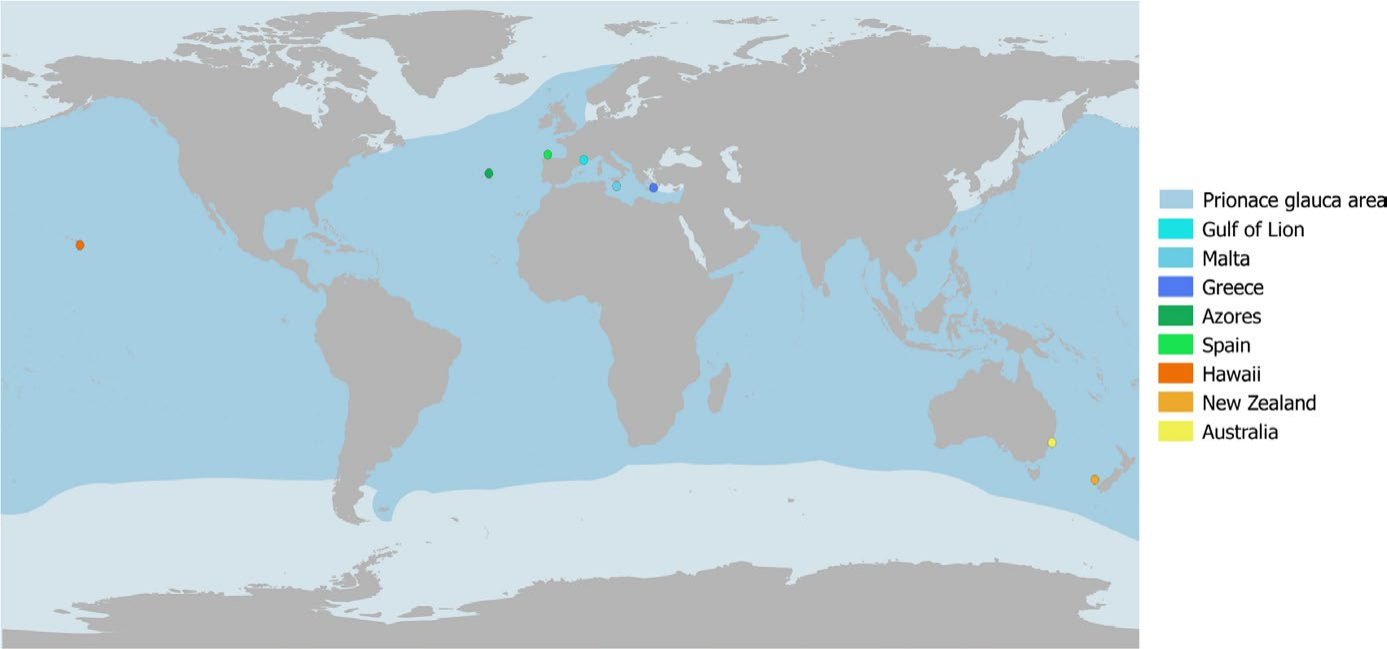
Sampling sites across blue shark distribution area. The distribution area is drawn in blue, and sampling sites are represented by blue dots for the Mediterranean Sea (Gulf of Lion, Malta and Greece), green for the Atlantic Ocean (Spain and Azores) and orange for the Pacific Ocean (Australia, New Zealand and Hawaii)

In this study, we analysed blue shark samples from Mediterranean Sea, North Atlantic and Pacific Oceans (Figure 2), using mitochondrial and nuclear DNA, to provide an initial assessment of genetic stocks across the range of the species. We then performed population simulations to generate in silico data illustrating the properties of the “population *grey zone*” and discuss their potential similarities with the in vivo results as obtained for blue sharks.

## 2 MATERIALS AND METHODS

### 2.1 Sample collections and DNA extraction

DNA was isolated from fin tissue or muscle samples collected between 2010 and 2014 in three oceanic basins (Table S1, Figure 2), mostly as bycatches from collaborative fisheries. Representative samples were collected in the Atlantic Ocean (Vigo, *Spain* and *Azores* islands), the Mediterranean Sea (*Gulf of Lion* including Grau du Roi and Corsica from France, *Malta*, and *Greece*) and the Pacific Ocean (*Hawaii*,* Australia* and *New Zealand*), the Indian Ocean could unfortunately not be sampled for this study. Samples were preserved in 96% ethanol and stored at room temperature.

DNA was extracted using CTAB (Doyle & Doyle, 1987) to which we added a digestion step using proteinase K.

### 2.2 Markers selection and PCR amplification

Genetic variation at two kinds of putatively neutral genetic markers (mitochondrial DNA/mtDNA and microsatellites) was used to test the hypothesis of population differentiation and bottlenecks. A fragment of the mitochondrial cytochrome *b* (cyt*b*) gene, 1,000 bp on average, was amplified with *Glu14314L (*5′‐CCAATAACTTGAAAAACTATCG‐3′) and *Thr15546H* (5′‐TCTTCGACTTACAAGGTC‐3′). Reaction volumes were 50 μl, containing 0.4 μl of *Taq* (at 5 U/μl), 5 μl of MgCl2 (at 25 mM), 5 μl of dNTP (at 8 mM), 5 μl of buffer (5×), 23.6 μl of pure water and 3 μl of each primer (at 10 μM). Cycling conditions were 94°C for 3 min, 35 cycles of 94°C for 30 s, 48°C for 1 min and 72°C for 1 min 15 s, finishing with a final extension at 72°C for 5 min. Paired‐end sequencing was performed using the classical Sanger method. The paired‐end sequences were assembled and aligned.

Ten microsatellites loci were used: A2ASY, BEF94, CY92Z, D0MST, DZOXN, EWU1E and FV6T5 (Taguchi et al., 2013), TB01, TB02 and TB04 (Mendonça et al., 2012). Except for TB01, loci were multiplexed (amplified and genotyped together) by sets of three loci using the “Type‐it microsatellite” kit by QIAGEN (QIAGEN GmbH, Hilden, Germany) in 25 μl reaction volumes containing 12.5 μl of Master Mix, 5.5 μl of pure water and 0.25 μl of each primer (at 10 μM). For TB01, amplifications were performed in 20 μl reaction volume containing 0.3 μl of *Taq* (at 5 U/μl), 2 μl of MgCl_2_ (at 25 mM), 2 μl of dNTP (at 1 mM total), 4 μl of buffer (5×), 6.1 μl of pure water, 0.4 μl for the reverse primer and 0.2 μl for the forward counterpart (both at 10 μM).

PCRs began with a hot start at 95°C for 5 min and continued as follows: for multiplexes—95°C for 30 s/63–57°C for 90 s/72°C for 30 s, for 35 cycles and 60°C for 30 min according to the QIAGEN protocol; for TB01, 95°C for 45 s/55–50°C for 45 s/72°C for 25 s, for 35 cycles and 72°C for 7 min. The PCR products were resolved using an ABI 3500xL (fluorescence‐based DNA analysis instrument using capillary electrophoresis technology with 24 capillaries from Applied Biosystems, Waltham, MA, USA) and scored manually.

The loci D0MST presented critical amplification failures and was eventually removed from data, leaving nine loci for further data analysis.

### 2.3 Analysis of genetic differentiation

For mitochondrial data, nucleotide (π) and haplotype (*h*) diversities (Nei, 1987) for each population were estimated with DnaSP 5.10.1 (Librado & Rozas, 2009). ARLEQUIN 3.1 (Excoffier, Laval, & Schneider, 2005) was used to estimate the genetic diversity and pairwise *F*_ST_ (Weir & Cockerham, 1984) as well as pairwise Phi_ST_ with 1,000 replicates for statistical *p*‐value computation. Adjustment for multiple comparisons was performed through *q*‐values as positive FDR (false discovery rate, Storey, 2003) computed with SGoF+ (Carvajal‐Rodriguez & de Uña‐Alvarez, 2011). An analysis of molecular variance (AMOVA) using a hierarchical approach setting oceanic basins as the first hierarchical level (Atlantic: Azores and Spain; Mediterranean: Gulf of Lion, Malta and Greece; and Pacific: Australia, New Zealand and Hawaii) was also performed to explore the population structure.

For microsatellite data, the expected heterozygosity (*H*_*E*_) under Hardy–Weinberg expectations, observed heterozygosity (*H*_*O*_) and estimators by Weir and Cockerham (1984) of Wright's *F*‐statistics were calculated using GENETIX 4.05 (Belkhir, Borsa, Chikhi, Raufaste, & Bonhomme, 1996–2004). The significance levels of *F*_*IS*_ and *F*_ST_ were assessed via 1,000 permutations. Allelic richness after rarefaction for the smallest entire sample size (*N* = 13, Australia) was calculated using FSTAT (Goudet, 1995). *F*_ST_ *p*‐values for multiple comparisons were also assessed using FDR by SGoF+.

We used the program POWSIM 4.1 from Ryman and Palm (2006) to determine the statistical power of our markers to detect genetic differentiation given differences in their levels of allelic diversity and sample sizes. As POWSIM computes Nei's *F*_ST_ (Nei, 1987; Nei & Chesser, 1983) “modified to be independent of the number of subpopulations” (POWSIM manual), we used GENETIX and mmod package 1.3.3 (Winter, 2012) implemented within the statistical software R 3.2.0 (R Core Team 2015) to determine the Nei's *F*_ST_ values for both mitochondrial and microsatellites data sets. POWSIM enables to determine with chi‐square and Fisher tests if the data set power to detect the corresponding *F*_ST_ is sufficient, that is, superior to 80%. The parameters of the Markov Chain were fixed to 10,000, 1,000 and 10,000 for, respectively, dememorizations (burn‐ins), batches and iterations per run, for a total of 1,000 runs each.

### 2.4 Clustering analysis

Population structure was investigated using two statistical approaches. First, STRUCTURE 2.3.4 (Pritchard, Stephens, & Donnelly, 2000) and STRUCTURE HARVESTER 0.6.94 (Earl & vonHoldt, 2012) were used to reveal the clustering, if existing, in the data set. Ten independent runs were performed on STRUCTURE for each assumed number of population(s) *K* = 1–8 under an admixture model. This model is consistent with the fact that blue sharks are able to migrate over long distances. All runs were executed with 50,000 burn‐in periods and 200,000 MCMC (Markov chain Monte Carlo) repetitions, using the eight regions defined earlier as prior information. STRUCTURE HARVESTER displays results allowing to assess *K*, the number of genetic populations that best fit the data, based on maximum likelihood (Evanno, Regnaut, & Goudet, 2005).

In addition, a discriminant analysis of principal components (DACP, Jombart, Devillard, & Balloux, 2010) was performed. DAPC is a multivariate analysis that integrates principal component analysis (PCA) with discriminant analysis to summarize genetic differentiation between groups (Jombart, 2008; (adegenet package v2.0.1, Jombart, 2008). Sampling location was used as prior. While STRUCTURE forms genetic clusters of individuals by minimizing departure from Hardy–Weinberg and linkage disequilibria, DAPC maximizes genetic separation among groups and minimizes variation within groups (Jombart et al., 2010), which may constitute a more accurate approach for species exhibiting potentially high gene flow.

A haplotype network aiming to construct the shortest possible tree of haplotypes was computed using Network 4.6.1.4 (Fluxus Technology Limited 2010). We chose the median‐joining (MJ) network algorithm and epsilon (the weighted genetic distance to the known sequences in the data set) set to 0 (Bandelt, Forster, & Röhl, 1999).

### 2.5 Effective population sizes and bottleneck tests

Fu and Li's D (Fu & Li, 1993) and Fu's Fs (Fu, 1997) were used to test for the occurrence of demographic changes such as expansion or bottlenecks.

Tests for evidence of genetic bottlenecks were performed using BOTTLENECK 1.2.02 (Cornuet & Luikart, 1996) in every region defined and in the global sample. Wilcoxon signed rank tests (most appropriate and statistically powerful for data set with a limited number of polymorphic loci, Maudet et al., 2002) were used to investigate microsatellite heterozygote excess and the allele frequency distribution test. Genetic bottlenecks reduce allelic diversity faster than heterozygosity (Nei, 1989). Consequently, populations exhibit an excess of heterozygosity with a greater number of microsatellite loci than predicted by chance until mutation–drift equilibrium is established (Cornuet & Luikart, 1996). The genetic differentiation between populations would be greater if these populations had suffered from a recent bottleneck. The test was performed under two assumptions for the mutation model: the pure stepwise‐mutation model (SMM) and two‐phase mutation (TPM, Di Rienzo et al., 1994) with 70% proportion of SMM in TPM, 30% variance for TPM and 1,000 replicates.

We used NeEstimator 2.01 (Do et al., 2014) to compute the *N*_*e*_ estimates for every population and for the whole population with the parameters suggested for low sample size (linkage disequilibrium method, with *p*‐value of .02 and 95% confidence interval estimation by parametric method).

### 2.6 Simulations

Simulations were performed to characterize the genetic differentiation among two increasingly diverging populations, in order to illustrate the number of generations required to overcome the “population *grey zone,*” depending on the variation of the two driving demographic parameters: effective population size and migration. We used simuPOP 1.1.7 (Peng & Amos, 2008) to simulate pairs of populations of randomly mating individuals (recombination rate: 0.01) with 10 loci and 10 allelic states each. The pairs of populations were initiated to be genetically similar (with identical allelic frequencies of 0.1). The simulations were conducted up to 5,000 generations. Every 50 generations, *F*_ST_ was computed. Then, twice 100 subsamples of 50 individuals were drawn from populations. The sub‐*F*_ST_ values were computed on these pairs of subsamples, and the sub‐*F*_ST_ significance was assessed by randomly re‐assorting the individuals in the subsamples and computing a simulated *F*_ST_ 1,000 times. The proportion of significant sub‐*F*_ST_ values was thus obtained by calculating the proportion of sub‐*F*_ST_ with *p*‐values less than or equal to .05. The *F*_ST_ values represent the “true values” computed on the entire in silico‐generated populations and the sub‐*F*_ST_ values are based on a realistic subsampling comparable to the one classically performed in natural populations. We ran simulations for effective sizes (*N*_*e*_) of 10,000 and 100,000 and 1,000,000 with per‐generation numbers of migrants (*N*_*e*_ *m*) of 0, 1 and 10. Considering variations in *N*_*e*_ *m* may also result from a change in *N*_*e*_ at constant *m* (Figure 2), simulations were also performed to illustrate the impact of drastic bottlenecks and the time lag resulting with our genetic indexes. We ran simulations for initial effective sizes (*N*_*e*_) of 1,000,000 and with per‐generation numbers of migrants (*N*_*e*_ *m*) of 100. At generation 500, we reduced effective sizes either to 100,000 (bottleneck of 90%) or to 10,000 (bottleneck of 99%). The migration rate (*m *= 0.0001) was kept constant in both scenarios. *F*_ST_, sub‐*F*_ST_ and proportion of significant sub‐*F*_ST_ were computed every 50 generations before the bottlenecks and every 20 generations after.

## 3 RESULTS

### 3.1 Genetic variability

From a total of 201 sequences (Table 1) of 758 bp, cyt*b* was variable for 25 polymorphic sites, leading to the discrimination of 22 distinct haplotypes. Haplotype (*h*) and nucleotide (π) diversities ranged from 0.62 to 0.86 (with the exception of Greece where only three individuals were sampled, *h* = 0.067) and from 0.17 to 0.26, respectively.

**Table 1:** Genetic diversity within each sampling location based on mitochondrial DNA

Population	cyt*b* samples	Haplotypes	*h*	π	*D*	*F*_*s*_
Gulf of Lion	63	13	0.618	0.258	−1.186	−4.156*
Malta	25	8	0.757	0.209	−0.046	−2.497
Greece	3	3	0.067	0.176	NA	1.061
Azores	39	10	0.752	0.220	−2.788	−3.310*
Spain	24	7	0.861	0.167	−0.973	−2.319
Hawaii	9	5	0.840	0.198	−0.264	−1.504
New Zealand	27	8	0.739	0.174	−2.113	−3.031*
Australia	11	5	0.830	0.173	−0.402	−1.396

From left to right: the number of sequences obtained (cyt*b* samples), the number of unique haplotypes per location (haplotypes), the haplotype diversity (*h*), the nucleotide diversity (π), *D* of Fu and Li (1993) and *F*_*s*_ of Fu (1997). Statistical significance: * for p‐values < .05, ** for p‐values < .01 and *** for p‐values < .001.

A total of 229 genotypes of nine microsatellites were obtained, with four missing loci or less (179 genotypes without missing loci, 28 with only one locus and 22, i.e., 10%, with two to four missing loci). No tendency to an unbalanced amount of missing loci was observed in relation to the geographic area where the samples were caught. The mean number of alleles per loci ranged from 3.78 for Greece to 12.11 for the Gulf of Lion (Table 2). Once standardized to consider the minimum number of samples taken (13 entire genotypes, Australia), the allelic richness did not exhibit differences among geographic regions (Table 2). Similarly, the expected and observed heterozygosity were comparable, ranging between 0.69 and 0.76 (Greece excluded). No *F*_IS_ values departed significantly from 0 (Table 2).

**Table 2:** Genetic diversity within each sampling location, based on nuclear DNA

Population	μsat samples	Mean alleles	Ar(3)	Ar(13)	*H*_*e*_	*H*_*o*_	*F*_is_
Gulf of Lion	73	12.111	3.845	8.108	0.760	0.745	0.028
Malta	32	10.778	3.840	8.116	0.756	0.738	0.041
Greece	3	3.778	3.778	NA	0.642	0.815	−0.073
Azores	44	11.889	3.830	8.088	0.759	0.769	−0.002
Spain	26	10.111	3.695	7.916	0.727	0.730	0.016
Hawaii	8	5.111	3.809	NA	0.692	0.724	0.054
New Zealand	27	9.667	3.670	7.530	0.730	0.728	0.022
Australia	16	7.889	3.776	7.563	0.732	0.741	0.023

From left to right: the number of genotypes obtained (“μsat samples” stands for microsatellite samples), the mean number of alleles per location (mean alleles), the allelic richness after rarefaction for the smallest sample size (Ar(3)) and for the second smallest sample size (Ar(13)), the expected (*H*_*e*_) and observed heterozygosity (*H*_*o*_) and the inbreeding coefficient (*F*_is_).

### 3.2 Population differentiation

**Figure 3:**
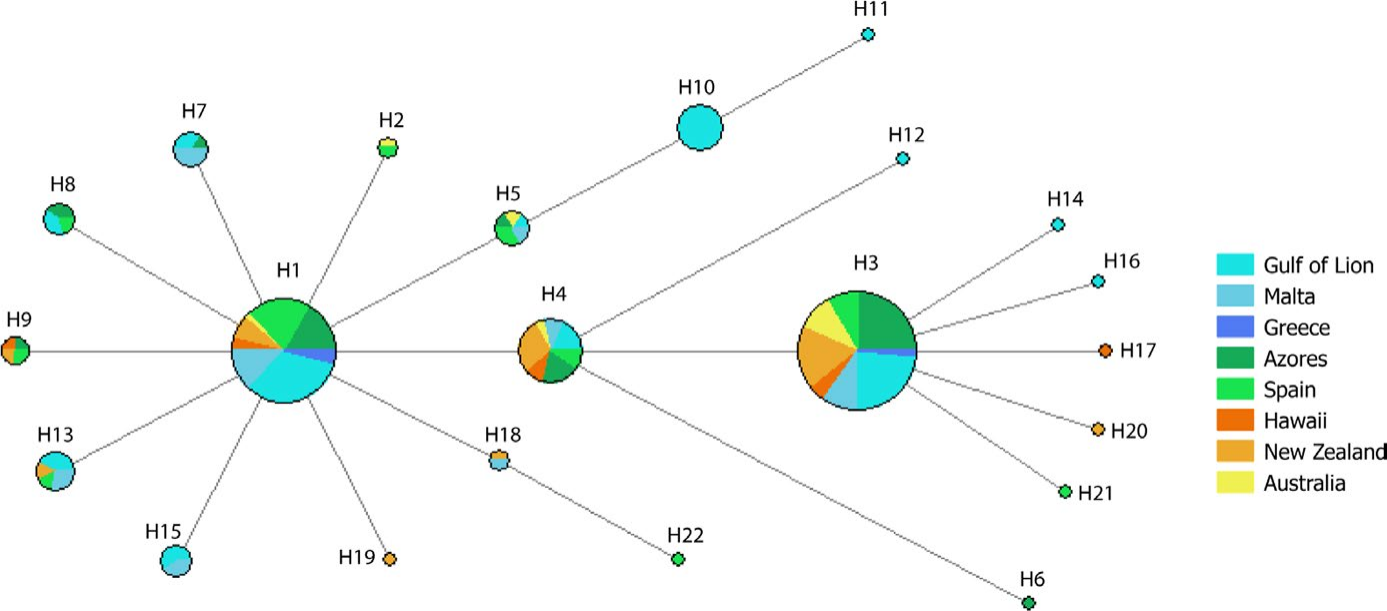
Haplotype network of blue shark individuals. Each circle represents a unique haplotype, and their sizes are proportional to the number of individuals sharing this haplotype. The colour inside each circle indicates the sampling site origin of the individual. The lengths of the branches joining the circles are proportional to the number of differences between the haplotypes

The haplotype network (Figure 3) showed three widely distributed major haplotypes and no spatial segregation of haplotypes. Similarly, the AMOVA showed no significant partition of the variance among the oceanic basins or among populations within oceanic basins.

Similar results were obtained through *F*_ST_ estimates based on both cyt*b* (Table 3a) and microsatellite data (Table 3b) and through Phi_ST_ estimates (Table S2). For cyt*b* (Table 3a), only *F*_ST_ between Australia and the Gulf of Lion was significant with a very low FDR (*p*‐value correction for multiple tests, *q*‐value = 5.84·10^−307^). As for microsatellites (Table 3b), the *F*_ST_ values showed no significant differentiation with a FDR lower than 0.05.

**Table 3:** Pairwise *F*_ST_ between sampling sites for mitochondrial (a) and nuclear (b) DNA

	Gulf of Lion	Malta	Greece	Azores	Spain	Hawaii	New Zealand
(a)							
Malta	−0.004						
Greece	−0.064	−0.078					
Azores	0.024	0.004	−0.035				
Spain	0.012	−0.002	−0.163	0.0312			
Hawaii	−0.008	−0.028	−0.061	−0.040	0.0007		
New Zealand	0.044*	0.020	0.040	−0.010	0.069*	−0.050	
Australia	0.084***	0.061	0.127*	−0.003	0.123**	0.014	−0.008
(b)							
Malta	−0.001						
Greece	0.021	0.013					
Azores	0.001	−0.003	0.021				
Spain	0.005	−0.0004	0.040	−0.001			
Hawaii	−0.010	−0.009	−0.015	−0.012	−0.007		
New Zealand	0.003	0.0001	0.024	0.007*	0.009*	−0.0123	
Australia	0.001	0.001	0.053*	0.002	0.008	−0.002	0.008

The significance of the *F*_ST_ values was assessed via 1,000 permutations: *for *p*‐values < .05, **for *p*‐values < .01 and ***for *p*‐values < .001. Once corrected for multiple tests (using the false discovery rate, Storey, 2003), only *F*_ST_ between Australia and the Gulf of Lion for cyt*b* (a) remained significant.

**Figure 4:**
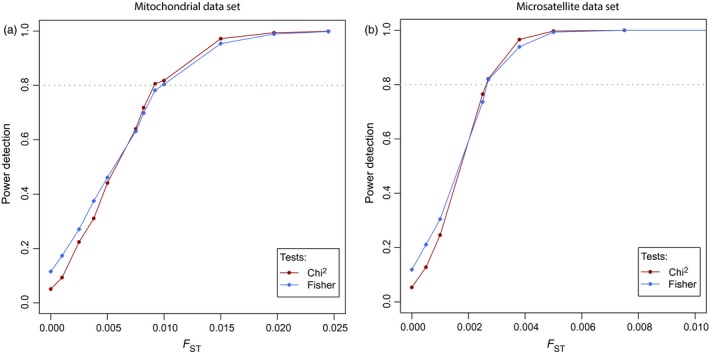
Power determination from mitochondrial (a) and microsatellite (b) data sets with POWSIM. Every couple of population size/number of generations corresponds to a *F*_ST_ value. POWSIM's results indicated the mitochondrial data set has the power to detect correctly *F*_ST_ values from approximately 0.01 (a) and the microsatellite data set *F*_ST_ values from approximately 0.0026 (b)

We chose to reproduce Eastwood, López, and Drew (2016) way of representing the results with 10,000 as population size and with an increasing number of generations of drift before sampling. The package mmod computed a Nei's *F*_ST_ of 0.0443 for mitochondrial data. POWSIM's results indicated the mitochondrial data set has the power to detect correctly *F*_ST_ values from approximately 0.01 (Figure 4a). GENETIX computed a Nei's *F*_ST_ of 0.0048 for microsatellite data. POWSIM's results indicated the microsatellite data set has the power to detect correctly *F*_ST_ values from approximately 0.0026 (Figure 4b). Results from POWSIM suggest data sets have the power to detect the corresponding genetic differentiations.

**Figure 5:**
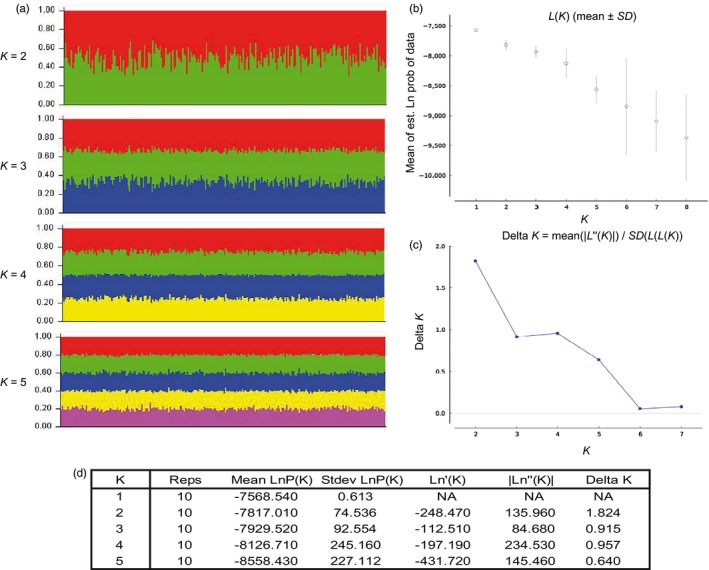
Bayesian clustering of blue shark individuals from STRUCTURE analysis. a: Within barplots for *K* from 2 to 5. Each individual is represented by a vertical bar partitioned into coloured sub‐bars whose lengths are proportional to its estimated probability of membership for the *K* clusters. b: Plot of the mean of estimated “log probability of data” for each value of *K*. c: Delta *K* of Evanno's method based on the rate of change in the log probability of data. d: Evanno table output for *K* from 1 to 5

In line with those results, whatever the k tested, STRUCTURE and STRUCTURE HARVESTER pictured a lack of structure (Figure 5). Comparable results were obtained with the DAPC analysis (Figure S1), where no distinct group emerged. In fact, no stark separation between locations was evident (Figure S1b) and only the three samples from Greece gave hints of difference along the first principal component (Figure S1a).

### 3.3 Effective population size and bottlenecks

Fu and Li's D values were highly negative but not supported (Table 1). Fu's Fs values were highly negative and supported for the Gulf of Lion, Azores and New Zealand (Table 1). Similarly, under the hypothesis of a SMM mutation model, a bottleneck was statistically supported in all groups of individuals except the ones from Australia (Table 4), whereas a bottleneck was detected only in the sample from Spain under the TPM model. NeEstimator could not provide an estimate for the upper 95% CI limit for any population (i.e., all upper limits were infinite), which may be due to large effective population sizes, or to the limited number of samples available to ascertain this estimate and the resulting lack of statistical power.

**Table 4:** Results of genetic bottleneck test based on nuclear DNA

Population	TPM *p*‐value	SMM *p*‐value
Gulf of Lion	.281	3.340 × 10^−4^
Malta	.524	.026
Azores	.294	.028
Spain	.004	.004
New Zealand	.279	.032
Australia	.468	.543

From left to right: Wilcoxon signed rank test *p*‐values on possible microsatellite heterozygote excess for the pure stepwise‐mutation model (SMM) and two‐phase mutation (TPM) with 1,000 replicates each.

### 3.4 Simulations

**Figure 6:**
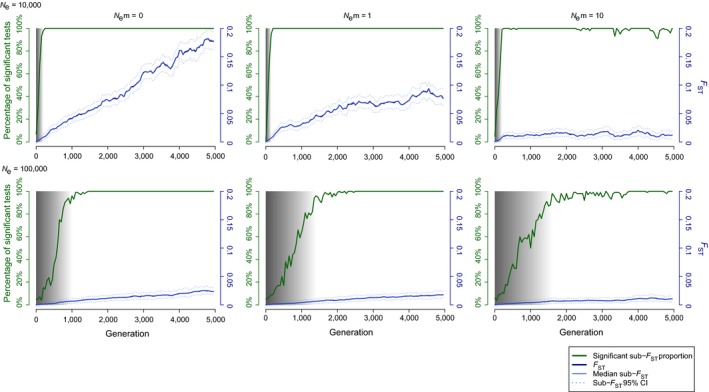
The “grey zone” of population differentiation illustrated with data from simulations of splits among populations with increasing size *N*_*e*_, exchanging a variable number of migrants *m*. Simulated population separation process with *N*_*e*_ = 10,000 and 100,000 and *N*_*e*_ *m* = 0, 1 and 10. For each plot, the *x*‐axis represents the number of generations since the divergence, the right *y*‐axis the *F*_ST_ values (blue lines, full for the median value and dashed for the 95% envelope) and the left *y*‐axis the percentage of significant *F*_ST_ values (green line). The “population *grey zone,*” in shades of grey, indicates the number of generations since the split, during which *F*_ST_ computed on subsamples is likely not to be statistically supported, and thus, the number of distinct populations will remain elusive

Whatever the effective size (*N*_*e*_) or the number of migrants exchanged (*N*_*e*_ *m*), the *F*_ST_ and the sub‐*F*_ST_ values are very similar (see Figure 6). The *F*_ST_ and sub‐*F*_ST_ values decrease with increasing number of migrants and effective sizes.

For an effective size of 10,000 individuals, the number of migrants exchanged has nearly no influence on the “grey zone” range: it takes an average of 200 generations to obtain a detection capacity of a significant sub‐*F*_ST_ of 95%. For an effective size of 100,000 individuals, the number of generations necessary ranges from at least 1,000 (*N*_*e*_ *m* = 0), to 1,400 (*N*_*e*_ *m* = 1), and to 1,600 (*N*_*e*_ *m* = 10).

**Figure 7:**
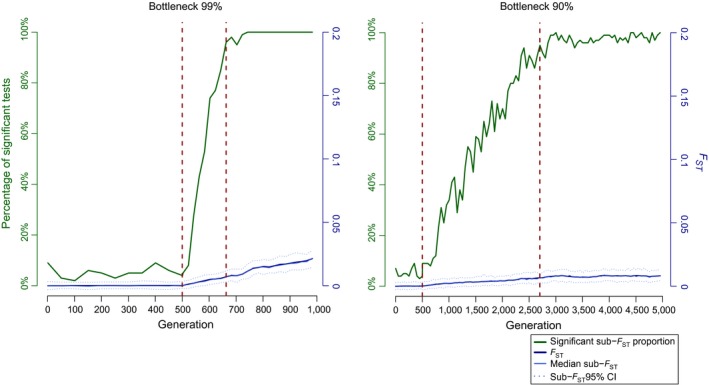
Illustration of the impact of huge bottlenecks with data from simulations of splits among populations. Simulated population separation process with initial *N*_*e*_ = 1,000,000 and *m* = 0.0001. After 500 generations (first dashed red line), each population was reduced by 90% and 99%. The detection of significant sub‐*F*_ST_ above 95% is indicated by the second dashed red line. For each plot, the *x*‐axis represents the number of generations since the divergence, the right *y*‐axis the *F*_ST_ values (blue lines, full for the median value and dashed for the 95% envelope) and the left *y*‐axis the percentage of significant *F*_ST_ values (green line)

As for the bottleneck results (Figure 7), the number of generations necessary to detect a significant change with the *F*_ST_ index ranges from at least 160 (bottleneck of 99%) to 2,200 generations (bottleneck of 90%).

Adding simulations with larger effective population sizes would be extremely computationally time‐consuming for an iterative process. The estimates given here are thus only lower bound in terms of generation time required for the divergence to be detected. The comparison of results obtained when switching effective population size thus delivers rather conservative estimates of the number of generations during which the “grey zone” effect applies. These results suggested such “grey zone” effect for effective population size of hundred millions individuals is likely even more pervasive then illustrated in Figure S2.

## 4 DISCUSSION

Widespread genetic homogeneity of one of the most widely distributed species worldwide, the blue shark, is supported here by the similar levels of allelic richness and lack of significant *F*_ST_ overall, in samples across the three oceanic basins included in this study (Mediterranean, Atlantic and Pacific). These results reflect large‐scale panmixia, that is, random mating at the worldwide scale, or a departure from random mating too recent to have left a signature on the genome, depending on the population size(s). Whether this genetic homogeneity reflects the existence of a single demographic entity and/or the demographic interdependence of all groups of individuals worldwide is essential information to manage the population(s). The low sample size and limited geographic coverage out of Europe, however, call for a cautious interpretation of results. Large‐scale panmixia is not entirely supported here for two reasons. First, the occurrence of a handful of significant pairwise *F*_ST_ values (with only one value left after correction for multiple tests), although low and distributed with no geographic coherence, does not allow to entirely reject the hypothesis of subtle pattern of very low genetic differentiation, difficult to grasp with the limited statistical power of our data set. Second, and more importantly, even in the presence of confirmed large‐scale panmixia based on a more robust data set, the scenario of widespread genetic interdependence can hide a wide range of demographic situations, as we will develop and illustrate below, leading us to propose the concept of “population *grey zone*.”

This lack of genetic differentiation at a wide scale is consistent with the recent reports of genetic homogeneity of this species at a regional scale in the Indo‐Pacific (Ovenden, Kashiwagi, Broderick, Giles, & Salini, 2009; Taguchi, King, Wetklo, Withler, & Yokawa, 2015) and in the North Pacific (King et al., 2015), and at broad scale between Atlantic North and South and between Atlantic and Pacific (Verissimo et al., 2017) and between Atlantic and Mediterranean Sea (Leone et al., 2017; but only with mtDNA). This result is as well consistent with the broad‐scale panmixia reported for other widely distributed species, such as lemon sharks (Feldheim, Gruber, & Ashley, 2001; Schultz et al., 2008), scalloped hammerhead sharks (*Sphyrna lewini*, Ovenden et al., 2011; Daly‐Engel et al., 2012), milk sharks (*Rhizoprionodon acutus*, Ovenden et al., 2011), school sharks (*Galeorhinus galeus*, Hernández et al., 2015) and basking sharks (*Cetorhinus maximus*, Hoelzel, Shivji, Magnussen, & Francis, 2006). Blue sharks are capable of long‐distance migrations (Vandeperre et al., 2014) and sperm storage as well as delayed fecundation (Carrera‐Fernández et al., 2010), life‐history traits shared with the species listed above, which likely contribute to the lack of any apparent strong barrier to gene flow among distant ocean basins. The few significant *F*_ST_ values, however, suggested at least very mild and randomly distributed pairwise departure from panmixia, as also observed in many of the other shark species exhibiting a similar pattern of large‐scale panmixia (Feldheim et al., 2001; Hernández et al., 2015; Ovenden et al., 2011). The *F*_ST_ estimates based on microsatellites support a limitation of gene flow between groups of specimens sampled in the Atlantic and the ones caught in New Zealand, but not the Australian ones, nor between the Atlantic and Mediterranean samples. The mitochondrial DNA shows hints of differentiation between the Pacific and Atlantic populations, suggesting a possible distinct pattern of migration of females (Vandeperre et al., 2014), or the existence of a restriction to gene flow too recent to be detected in large effective population sizes but easier to notice in mtDNA markers with comparatively reduced *N*_*e*_. However, again, these differentiations are neither systematic nor entirely coherent (Table 3a,b), rendering their interpretation in terms of population structure and management speculative and supporting the need for more robust analysis to ascertain the existence of differentiated stocks. When our individuals were grouped per sampling locations, all (under the SMM mutation model) exhibited significant results at the bottleneck test. Given the limitations for the interpretation of those tests, based on microsatellites, such reduction may have occurred in very ancient (last glaciations) as well as very recent (20th century) times. Three hypotheses may explain this rather homogeneous bottleneck. Either (i) the genetic bottleneck is ancient (and thus, blue sharks may form only one population, but also may form several populations having shared the same or a similar environmental pressure(s) in the past) or (ii) the genetic bottleneck is recent (and thus, blue shark population is likely panmictic, suffering rather uniformly a present‐day reduction in population size) or (iii) a combination of both resulting in bottlenecks detection without population differentiation. The capacity of next‐generation sequencing to access high‐density genome scanning is expected to allow improved inference of parentage or kinship through coalescent analyses, to expand analyses based on linkage disequilibrium (Hellberg, 2009) and to refine both the dating of bottlenecks and the interpretation of patterns of genetic differentiation (Waples, Seeb, & Seeb, 2016). For top predators such as the blue shark, the reconstruction of genealogies through genome scanning and the use of linkage disequilibrium may facilitate a better understanding of even low patterns of restriction on gene flow, if they exist.

Importantly, if large‐scale panmixia is confirmed by further genetic analysis, this case study exemplifies a frequent problem in interpreting population genetics data to understand demography and/or feed possible management strategies. Patterns of significant genetic differentiation can be interpreted, if at equilibrium, as reflecting both the genetic independence and demographic independence of the identified populations, helping to delineate the existing stocks, whereas genetic panmixia, even if confirmed, does not equate to demographic unity. In this case, tag studies would support the existence of two blue shark stocks in the North (Kohler & Turner, 2008) and South Atlantic (da Silva, Kerwath, Wilke, Meÿer, & Lamberth, 2010), consistent with the different periods of reproduction that may suggest a phase difference among populations inhabiting distinct hemisphere. Similarly, despite the apparent homogeneity of the Mediterranean and Atlantic groups of specimens, no (Queiroz et al., 2005, 2016; Vandeperre et al., 2014) or very rare (Kohler & Turner, 2008) shark movements were detected between the Atlantic and Mediterranean basins using conventional tagging. This result may be due to the low statistical power and representativeness of tags on a limited number of specimens but also could reflect a real demographic independence not revealed in the genetic estimates of differentiation.

Indeed, the particular life‐history traits of several marine species (large population sizes and high dispersal potential) often lead to weak or no genetic differentiation (Hedgecock et al., 2007; Waples, 1998), which could be explained by an homologous version at the intraspecific level (Figure 1) of the “species grey zone” concept proposed by De Queiroz (2007), that would be here the “populations *grey zone*.” We represented this concept through simulations (Figure 6). Figure 6 presents the evolution of *F*_ST_ over time since the split of populations, as well as the associated probability of rejecting the null hypothesis of panmixia, depending on effective size (*N*_*e*_) and the per‐generation number of migrants (*N*_*e*_ *m*). In the absence of genetic split of populations (i.e., null values of *F*_ST_, genetic panmixia), the rate of per‐generation number of migrants (*m*) ensuring genetic homogeneity may be largely insufficient to lead to demographic interdependency or to ensure a rescue effect (Gagnaire et al., 2015; Waples & Gaggiotti, 2006). Within the “population *grey zone,*” the pace of drift and thus the accumulation of detectable genetic differentiation depend strongly on the population size and number of migrants (Figure 6). Thus, despite migration rates low enough to ensure demographic interindependence and contribute to the accumulation of divergence of populations in terms of allelic frequencies, the detection of population differentiation may not be possible for many (thousands of) generations. Only after the sufficient number of generation elapsed to exit the “population *grey zone*” will the systematic rejection of panmixia allow the safe conclusion of demographic independence.

During the last glacial episode, colder water conditions may have caused a cessation of between‐ocean gene flow for the blue shark, a temperate species. Verissimo et al. (2017) reviewed the cosmopolitan coastal pelagic carcharhinoids and oceanic epipelagic sharks for which isolation and lineage divergence have been shown between Atlantic and Indo‐Pacific, probably due to colder water conditions around the tip of South Africa and to the cold Benguela current. These authors concluded that the current apparent genetic homogenization of the species is due to extensive interbasin gene flow since the last glacial period, which may apply the blue shark. However, according to simulations presented here, apparent panmixia is also compatible with the opposite scenario of a limitation to gene flow following the reorganization of the distribution range, nursery and feeding grounds after the end of the last glaciations. Considering the estimated generation time of blue sharks (8.1 years, IOCT 2007), the time required to escape the “grey zone” of detection corresponds to 12,960 years with *N*_*e*_ = 100,000 (Figure 6). Such effective population size is rather conservative considering the estimated 20 million individuals extirpated yearly by fishing for this species, although recent genetic‐based estimates suggested effective population sizes of only several thousand individuals in the Atlantic (Verissimo et al., 2017) and Pacific (King et al., 2015). In fact, within the hypothesis of the “grey zone,” even a number of migrants per generation of 10 with effective population size of 100,000 individuals are enough to hide until nowadays a genetic divergence initiated about 11,500 years ago.

The simulations performed here to test for the effect of a variation in *N*_*e*_ *m* driven by a reduction in *N*_*e*_ rather than a modification of *m* also showed an extensive time lag (Figure 7). The removal of 99% of a population of high effective size results in significant *F*_ST_ only 160 generations at least after the occurrence of the demographic event. Expectedly, less severe bottleneck expands further the genetic lag effect (Figure 7). These results imply that the 97% decline in abundance of the blue shark in the Mediterranean Sea during the mid‐20th century (Ferretti et al., 2008) is fully compatible with the lack of structure detected by our analysis, as it may only result in significant *F*_ST_ after 1,300–17,820 years depending on the strength of the bottleneck suffered.

This “population *grey zone*” can also be suspected and illustrated with other species, including at least the wahoo (*Acanthocybium solandri*, Theisen, Bowen, Lanier, & Baldwin, 2008) and the white marlin (*Kajikia albida*, Mamoozadeh, McDowell, Rooker, & Graves, 2017). The “population *grey zone*” may well explain as well the rather elevated number of studies where the null hypothesis of panmixia could not be rejected despite other data supporting the existence of distinct stocks. For example, the lack of genetic differentiation between dolphinfish (*Coryphaena hippurus*) individuals of Indo‐Pacific and Atlantic could be explained by the hypothesis of recent dispersal as proposed in Díaz‐Jaimes et al. article (2010) but also by the “population *grey zone*” theory. Despite limited dispersal supported by tag studies and the definition of two stocks based on morphologies and tags, no genetic structuration was observed for the sailfish (*Istiophorus platypterus*) in the Atlantic (McDowell & Graves, 2002). The “population *grey zone*” also supports previous warnings against the interpretation of genetically homogeneous populations as reflecting the occurrence of a single demographic entity. For example, Ely et al. (2005) study found no genetic differentiation between nor within oceanic basins for skipjack tuna (*Katsuwonus pelamis*) while Kumar and Kocour review (2015) reports several stocks/populations revealed by more recent studies with different mtDNA regions. Finally, the apparent panmixia between Atlantic and Indian Ocean populations of albacore (*Thunnus alalunga*) recorded with microsatellites (Montes et al., 2012) was recently demonstrated with SNP as hiding substructuration (Laconcha et al., 2015).

Beyond the “population *grey zone*” concept, it is also important to keep in mind that for numerous species supposed to exhibit specific geographic grouping of some specific life cycle or during particular seasons, through behaviour such as female philopatry (or pupping site for blue shark, Bustamante & Bennett, 2013), an a priori on the groups of samples susceptible to form (sub)populations is difficult to make on the basis only of their catch area. The use of exploratory algorithms as clustering methods or principal component analyses may (although partially) balance the usually opportunistic sampling strategies (due to logistic constraints or to the lack of knowledge of areas such as breeding areas, spawning grounds and nursery areas). Classical paired‐*F*_ST_ computed on groups of individuals sampled in an opportunistic way and then regrouped artificially according to the capture area is indeed less satisfying. As stated by Graves and McDowell (2015), the opportunistic sampling limited the power of genetic studies to elucidate population structuring for many billfish species. These authors recommended that, aside from larger sample sizes of individuals and molecular markers, “the development of biologically meaningful sampling designs that incorporate information on the movement patterns and life histories of these pelagic fishes.” For example, the genetic stock assessments for Atlantic bluefin tuna (*Thunnus thynnus*) mostly showed no structure until Carlsson, McDowell, Carlsson, and Graves (2007) analysed young‐of‐the‐year specimens near the spawning grounds and detected population structure. Contrastingly, Verissimo et al. (2017) sampled young‐of‐the‐year and small juvenile blue sharks in three Atlantic nurseries (Azores, South Africa and western Iberia) and found basinwide panmixia.

The effective management of fisheries requires a clear definition and understanding of the stock structure of the target species (Begg & Waldman, 1999), and this difference between the genetic and demographic concept of populations parallels the problem of the multiple existing definitions of stocks. Booke (1981, in Waples & Gaggiotti, 2006) defined a stock as a species, group or population of fish that maintains and sustains itself over time in a definable area. Although informative from a fishery perspective, this definition does not encompass the concept of the interdependence or independence of groups of individuals and thus does not provide useful information for the management and sharing of common resources. Depending on whether the perspective and scale at stake are genetic and evolutionary, demographic and ecological or management, two more operational and complementary definitions of stock may be considered, each corresponding to one of the two main concepts of connectivity described above. The genetic stock, according to Ovenden (1990), defines a reproductively isolated unit, which is genetically different from other stocks (genetic connectivity), while the harvest stock (Gauldie, 1991) designates a “locally accessible fish resource in which fishing pressure on one resource has no effect on the abundance of fish in another contiguous resource” (demographic connectivity). A stock thus might be an aggregate of biologically homogeneous (according to Booke's terms) but genetically different groups (Ovenden, 1990) or “substocks” (Altukhov, 1981), characterized by differentiated gene pools and/or demographically independent (according to Gauldie's definition) groups. From a stock management perspective, Gauldie's definition would be the most accurate, whereas from a longer‐term conservation perspective, recognizing and preserving potentially differentiated gene pools is also essential (Laikre, 2010). It requires, however, a rigorous sampling strategy whenever possible, and a clear account for the “population *grey zone*” concept.

## 5 CONCLUSIONS

Elasmobranch recovery is possible but requires in situ management actions (Ward‐Paige, Keith, Worm, & Lotze, 2012). Nursery protection has been proposed (Beck et al., 2001), as juvenile survival seems to be key for shark conservation (Cortés, 2002). However, the benefits of marine‐protected areas for mobile species are questioned (Grüss, Kaplan, Guénette, Roberts, & Botsford, 2011), even more so when fishery fleets would not respect them (Baum et al., 2003; Botsford, Castilla, & Peterson, 1997). As putative nurseries in the Atlantic, Mediterranean and Pacific overlap with tuna vessels and swordfish fleets, creating protected areas may be unrealistic and/or unproductive. Most of the time, blue sharks are not even targets but mere bycatch. A recent study (Queiroz et al., 2016) showed an 80% overlap between pelagic sharks' hot spots, including blue sharks, and longline fleets in the North Atlantic. In the absence of prospects for successful marine‐protected areas, the management of fish stocks is essential and requires a good knowledge of their delineation. As a long‐living and slow‐growing species, the blue shark is vulnerable to fisheries, which could rely on stock production models defined on traditional short‐living species and the recovery time estimated with fishery management plan greatly underestimated (Musick, 1999). Effective mitigation measures exist to reduce elasmobranch bycatch mortality for fisheries, if employed (Poisson et al., 2016).

The case study used here to illustrate the “population *grey zone*” does not reject the possibility that the blue sharks all belong to a single worldwide population. On the one hand, such global panmixia may fit a very large global population size, providing robustness to the species in cases of local depletion. On the other hand, depending on the spatial dynamics of the species, it may also imply that any serious impact in any part of the world might have serious consequences at a global scale. In both cases and as suggested by Theisen et al. (2008) who reported similar results and wonders on the pelagic wahoo, such a global population call for a global management. The wide‐scale decline or extirpation of such a top predator as blue sharks is a serious concern (Ferretti, Worm, Britten, Heithaus, & Lotze, 2010; Lewison, Crowder, Read, & Freeman, 2004; Myers, Baum, Shepherd, Powers, & Peterson, 2007; Rogers & Ellis, 2000; Stevens, Bonfil, Dulvy, & Walker, 2000). A conservative, concerted and global management and conservation strategy is thus required until in‐depth analysis allows the confirmation of homogeneity or delineation of differentiated demographic groups and/or stocks. The complex relationship among populations could be resolved by more powerful high‐density genome scan analysis. The scoring of thousands of genetic markers, allowing the identification of outlier loci, has proven useful for delineating local stock and defining conservation units (Nielsen, Hemmer‐Hansen, Larsen, & Bekkevold, 2009), as well as for the reconstruction of pedigrees to estimate stock size and number (Bravington, Grewe, & Davies, 2014). All emerging methods allowing the high‐density coverage of the genome are prospects to overcome the “population *grey zone*” problem likely responsible for the frequent mismatch between high expectations and inconclusive results obtained thus far when applying population genetics to detect differentiated stocks of fisheries' targets.

## DATA ARCHIVING STATEMENT

Data available from the Dryad Digital Repository: https://doi.org/10.5061/dryad.k302g
