# Supplementary figures and images for: Large‐scale genetic panmixia in the blue shark (Prionace glauca): A single worldwide population, or a genetic lag‐time effect of the “grey zone” of differentiation?
Source: Evol Appl. 2018 Feb 22;11(5):614–30. doi: 10.1111/eva.12591 (PMC5978958; doi:10.1111/eva.12591)

(a)

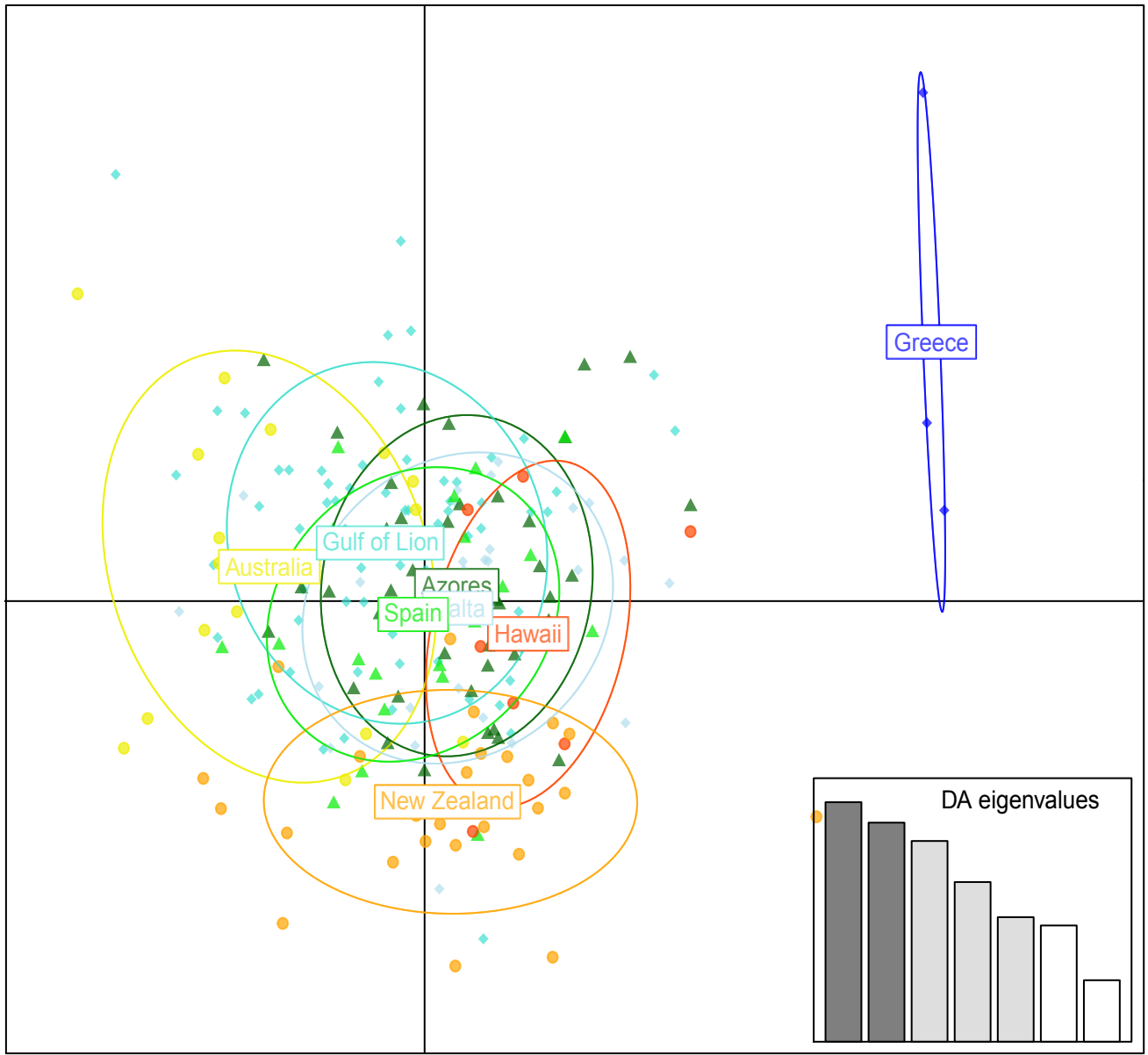

(b)

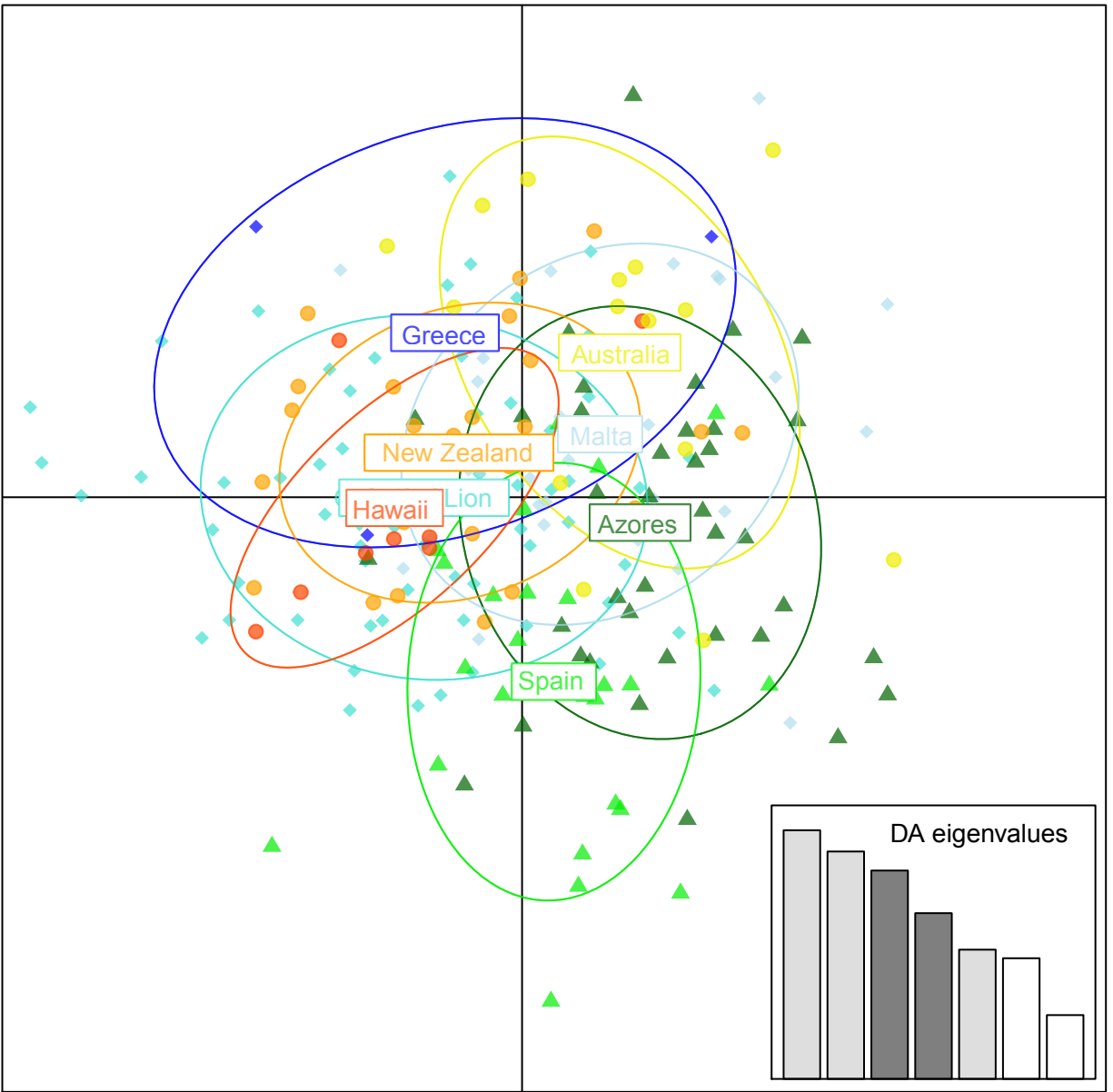

Supplement: Supplementary file 1 [file EVA-11-614-s001.pdf]

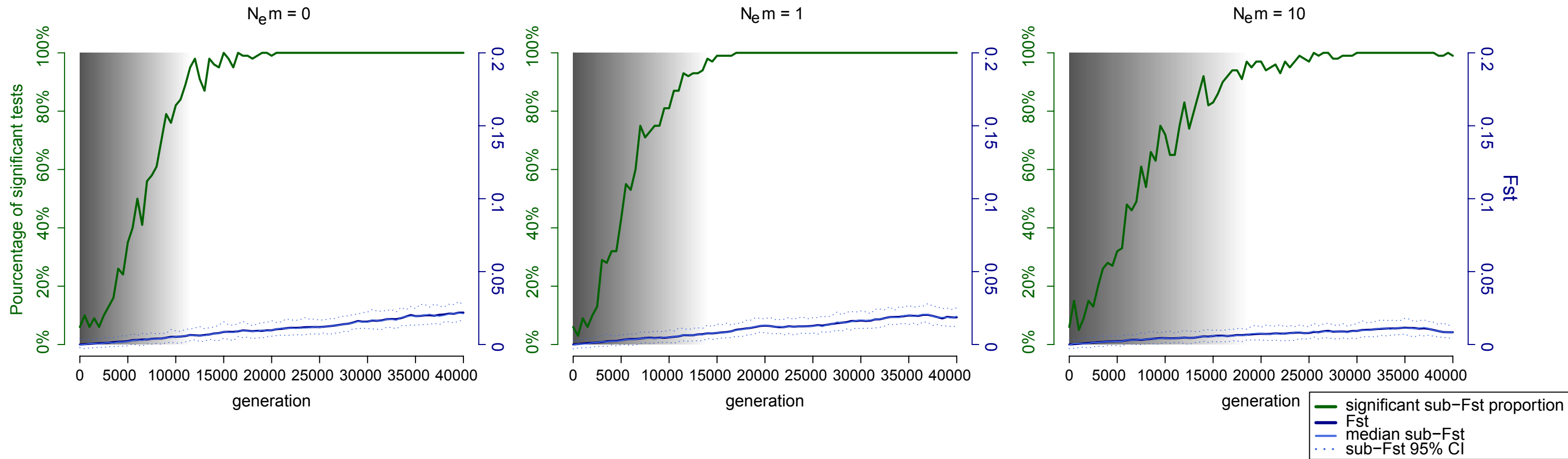

Supplement: Supplementary file 2 [file EVA-11-614-s002.pdf]
