# Supplementary material for: Large‐scale genetic panmixia in the blue shark (Prionace glauca): A single worldwide population, or a genetic lag‐time effect of the “grey zone” of differentiation?
Source: Evol Appl. 2018 Feb 22;11(5):614–30. doi: 10.1111/eva.12591 (PMC5978958; doi:10.1111/eva.12591)
Supplement: Supplementary file 3 [file EVA-11-614-s003.docx]

**Supplementary Information**

**Table S1:** Sampling site locations.

| Population | Long_GPS | Lat_GPS | samples |
| --- | --- | --- | --- |
| Gulf of Lion | 42.9964 | 4.0003 | 81 |
| Malta | 35.9375 | 14.3754 | 45 |
| Greece | 35.4855 | 26.1071 | 3 |
| Azores | 39.3582 | -26.0585 | 49 |
| Spain | 44.5000 | -7.4166 | 26 |
| Hawaii | 19.8968 | -155.5828 | 9 |
| New Zealand | -43.6500 | 165.8333 | 30 |
| Australia | -33.5740 | 152.2667 | 16 |

For each sampling site, the mean longitude (Long_GPS) and latitude (Lat_GPS) and the number of blue sharks sampled.

**Table S2**: Pairwise *Phi_ST_* between sampling sites for mitochondrial DNA.

|  | Gulf of Lion | Malta | Greece | Azores | Spain | Hawaii | New Zealand |
| --- | --- | --- | --- | --- | --- | --- | --- |
| Malta | 0.00807 |  |  |  |  |  |  |
| Greece | -0.15538 | -0.19760 |  |  |  |  |  |
| Azores | 0.05812** | 0.03727 | -0.14019 |  |  |  |  |
| Spain | -0.00018 | -0.01964 | -0.20840 | 0.04670 |  |  |  |
| Hawaii | 0.04711 | 0.03570 | -0.13793 | -0.05225 | 0.05996 |  |  |
| New Zealand | 0.09411** | 0.08308* | -0.06349 | -0.01471 | 0.10734* | -0.05816 |  |
| Australia | 0.10132* | 0.11887* | -0.01155 | -0.01292 | 0.15010* | -0.04426 | -0.02840 |

The significance of the *Phi*_ST_ values was assessed via 1000 permutations: * for p-values < 0.05, ** for p-values < 0.01 and *** for p-values < 0.001. Once corrected for multiple tests (using the False Discovery Rate, Storey 2003), no *Phi_ST_* remained significant with a q-value bellows 0.05.
